# Insulinome associé à une insuffisance corticotrope et un hypogonadisme hypergondadotrope: à propos d’une observation

**DOI:** 10.11604/pamj.2019.34.32.14865

**Published:** 2019-09-16

**Authors:** Faten Hadjkacem, Mahdi Kalthoum, Dorra Ghorbel, Mouna Ammar, Mouna Elleuch, Nadia Charfi, Mouna Mnif, Mohamed Abid

**Affiliations:** 1 Service d’Endocrinologie et Diabétologie de CHU Hédi Chaker, route El-Ain, 3029 Sfax, Tunisie

**Keywords:** Hypoglycémie, insulinome, insuffisance corticotrope, hypogonadisme, Hypoglycaemia, insulinoma, adrenocorticotropic deficiency, hypogonadisms

## Abstract

L’insulinome pancréatique est une tumeur neuroendocrine rare, souvent bénigne, mais qui peut mettre en jeu le pronostic vital du fait des accidents hypoglycémiques qu’elle engendre. L'insuffisance surrénalienne peut aussi être responsable d'hypoglycémie. Nous rapportons le cas d'un patient de 68 ans hospitalisé pour des hypoglycémies récurrentes, les explorations ont montré un hyperinsulinisme endogène, une insuffisance corticotrope et un hypogonadisme hypergondadotrope. Le patient était substitué par hydrocortisone et devant la non amélioration de la symptomatologie le diagnostic topographique d'insulinome était retenu 5 ans plus tard. On discute dans cet article les aspects cliniques, biologiques, radiologiques et thérapeutiques de l'insulinome ainsi que son rôle dans la genèse d'insuffisance corticotrope et d'hypogonadisme périphérique.

## Abstract (en)

Pancreatic insulinoma is a rare, often benign, neuroendocrine tumor which may give rise to life-threatening consequences due to hypoglycemia-related accidents. Adrenal deficiency can also cause hypoglycemia. We report the case of a 68-year old patient hospitalized for recurrent hypoglycaemia. Tests were performed that showed endogenous hyperinsulinism, adrenocorticotropin deficiency and hypergonadotropic hypogonadism. The patient received hydrocortisone without improvement. Five years later topography showed insulinoma. This study highlights the clinical, biological, radiological and therapeutic features of insulinoma as well as laboratory test results and shows that insulinoma can cause adrenocorticotropic deficiency and peripheral hypogonadism.

## Introduction

L’insulinome est l’une des tumeurs neuroendocrines les plus fréquentes du pancréas (55%). Il reste cependant rare avec une incidence de moins de 5 cas par millions d’habitants par an [1, 2]. Il est défini par une prolifération des cellules Béta des ilots de Langerhans, responsable d’une sécrétion excessive et inadaptée d’insuline entrainant des accidents hypoglycémiques. Cette tumeur peut survenir à n’importe quel âge avec une prédilection pour la 5^ème^ décennie. Elle touche de façon presque égale les deux sexes avec parfois une prédominance féminine [3-6]. La tumeur est unique et bénigne dans 90% des cas [5]. Dans 10% des cas, elle est multiple et s’intègre souvent dans le cadre des Néoplasies Endocriniennes Multiples de type I (NEM I). Son siège est presque exclusivement pancréatique. Cliniquement, l’insulinome donne des hypoglycémies, parfois très épisodiques, chez des adultes souvent jeunes et bien portants. Les signes de neuroglycopénie sont au premier plan, souvent mal rapportés par le patient. Ce qui explique le délai généralement long entre les premiers symptômes et la confirmation diagnostique. Le diagnostic positif est souvent aisé et se base sur les données biologiques qui confirment l’hyperinsulinisme endogène par le dosage combiné de la glycémie, de l’insulinémie et du peptide C. Le diagnostic topographique peut s’avérer difficile du fait de la petite taille de la tumeur échappant ainsi aux explorations radiologiques et parfois même à l’exploration peropératoire. Nous rapportons le cas d'un patient âgé de 68 ans chez qui l'exploration des hypoglycémies confirmait une insuffisance corticotrope initialement et un insulinome 5 ans plus tard.

## Patient et observation

Il s’agit d’un patient âgé de 68 ans, hospitalisé pour exploration de malaises hypoglycémiques récidivants. Il avait comme antécédent personnel une hypertension artérielle depuis 3 ans équilibré sous captopril 50 mg/j. L'histoire de sa maladie remontait à 2 ans marquée par l'installation de malaises survenant à raison de 3 fois par semaine à jeun et en post-prondial, faites de signes adrénergiques (asthénie, sueurs, palpitation) et parfois de signes neuroglycopéniques (céphalées, agitation, perte de connaissance). Ces crises s’amélioraient après resucrage. Une glycémie à jeun demandée était basse à 2.14 mmol/l. Devant la répétition des crises le patient était confié pour exploration et prise en charge.

L’interrogatoire avait éliminé la notion de prise médicamenteuse de toxique hypoglycémiant ou d’alcool. L'examen physique à l’admission trouvait un patient en bon état général. L’indice de masse corporel était à 23 kg/m^2^. La glycémie au doigt était de 1.12g/l. Il était en euthyroïdie clinique avec thyroïde non palpable et il présentait quelques signes d’insuffisance corticotrope: asthénie qui s’aggrave au cours de la journée associée à un jeun mal toléré à cause des malaises hypoglycémiques. Il avait une dépigmentation des aréoles et une tension artérielle basse à 90/60 mmHg alors que ses chiffres antérieurs étaient élevés. Pour l’axe gonadotrope, il signalait une dysfonction érectile avec baisse de la libido associé à une diminution de la fréquence de rasage et à l’examen il avait une gynécomastie minime unilatérale gauche et des organes génitaux externes types masculins adultes. Le reste de l’examen somatique était sans anomalies.

Sur le plan biologique, il avait une fonction rénale et hépatique normale. Au cours de son hospitalisation, le patient avait présenté deux malaises d’hypoglycémie symptomatiques et les glycémies veineuses concomitantes à ces malaises étaient de 1,7 mmol/l et de 2,75 mmol/l. Un dosage de l’insulinémie au moment de ces hypoglycémies était réalisé confirmant l’hyperinsulinisme devant une insulinémie élevée à 27,2 mUI/l et à 30,2 mUI/l respectivement. L’indice de Turner (IT) calculé était élevé à 544 au cours du premier malaise et à 151 lors de la deuxième crise hypoglycémique. Le dosage du peptide C lors du premier malaise était élevé à 4,13μg/l confirmant alors le caractère endogène de l’hyperinsulinisme. Devant ce tableau, on avait éliminé l’hypoglycémie factice par la prise de médicament insulino-sécréteur et les hypoglycémies auto-immunes par la négativité des anticorps anti insuline (Ac anti-insuline < 0,4 UI/ml). À la recherche d’un insulinome on avait complété par un scanner pancréatique qui était normal. L’écho endoscopie pancréatique n’avait pas été réalisée pour notre patient. Il s’agissait donc d’un tableau d’hypoglycémie organique chez un patient de 68 ans dont l’enquête biologique avait conclu à un hyperinsulinisme endogène avec un diagnostic de localisation qui restait négatif.

Devant la suspicion d’insuffisance corticotrope, un test au synacthène 1μg sur cortisol était pratiqué et avait confirmé l’insuffisance surrénalienne devant l’absence de stimulation de cortisol (T30=177ng/ml et T60=140ng/ml). L’Adreno CorticoTrophic Hormone (ACTH) basse à 9,3pg/ml confirmait l’origine centrale de l’insuffisance surrénalienne. Le reste de l'hypophysiogramme objectivait un hypogonadisme hypergonadotrope (testostérone: 1,7ng/ml, FSH: 45mUI/ml, LH: 26,5mUI/ml). Par ailleurs, l’axe lactotrope, somatotrope et thyréotrope était épargné. L’imagerie par résonance magnétique (IRM) de la région hypothalamo-hypophysaire n’avait pas montré d’anomalie hypophysaire ni d’atteinte de la tige pituitaire.

L’enquête étiologique de l'hypogonadisme hypergonadotrope était négative: à l’interrogatoire on ne retrouvait pas la notion de traumatisme testiculaire ou d’orchite, également pas de traitement par radiothérapie ou chimiothérapie et pas d’exposition professionnelle à des toxiques. L’échographie testiculaire était sans anomalie et le caryotype avait montrait une formule chromosomique 46, XY. Le patient était traité par hydrocortisone 20mg/j. L’évolution était marquée par la diminution de la fréquence des hypoglycémies devenant très rares et la disparition des signes neuroglycopéniques.

**Figure 1:**
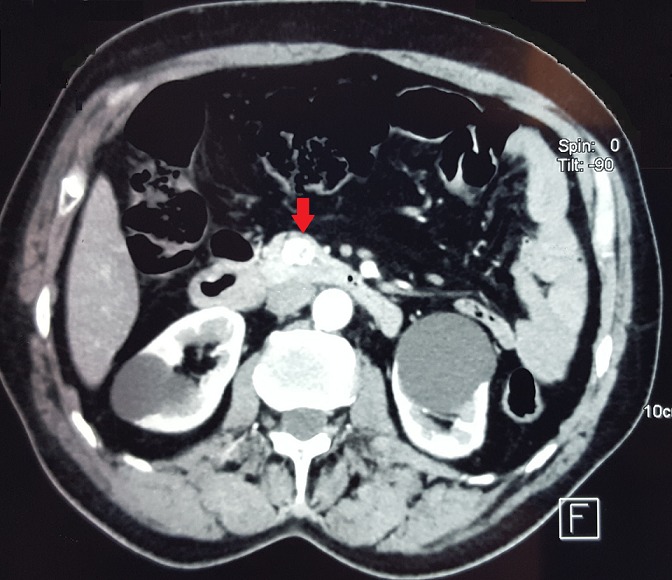
TDM (tomodensitométrie) pancréatique: lésion nodulaire bien limitée au niveau du pancréas de Winslow mesurant 16 x 15 mm qui se rehausse de façon intense au temps artériel

Après 5 ans et devant la réapparition des malaises hypoglycémiques chiffrés à 0,38 et 0,5g/l et la perte de conscience le patient était hospitalisé à nouveau au service d’endocrinologie. À l’interrogatoire, on retrouvait une prise de poids de 7Kg en 5 ans dont 3Kg les 2 derniers mois. L'exploration avait confirmé de nouveau l’hyperinsulinisme endogène (glycémie veineuse: 0,26g/l, insulinémie: 23,8mUI/l, IT: 595 et peptide C: 4,48μg/l). Une TDM pancréatique avait mis en évidence une lésion nodulaire bien limitée au niveau du pancréas de Winslow mesurant 16 x 15mm présentant un rehaussement intense et homogène au temps artériel (Figure 1).

L'IRM confirmait l’aspect d'une tumeur neuroendocrine de l'uncus du pancréas avec des multiples lésions hépatiques évoquant des shunts artério-veineux. Le diagnostic d'insulinome de pancréas était retenu et le bilan d'extension était négatif notamment la scintigraphie osseuse. Le patient avait bénéficié sous couverture par hémisuccinate d’hydrocortisone d’une énucléation de la tumeur et une cholécystectomie était aussi pratiquée. L’examen anatomopathologique avait montré un nodule pancréatique de 15 x 12mm dont l’immunohistochimie était fortement positive pour la chromogranine et CD56. Il s’agissait d'une tumeur endocrine bien différencié (grade I selon l'OMS).

L’évolution en post opératoire était marquée par la disparition des malaises hypoglycémiques et une perte de poids de 6kg associée à une normalisation du bilan biologique. En effet, la glycémie à jeun était de 4,76mmol/l; l’insulinémie à 4.2 mUI/l et un IT à 13,6. Le test synacthène 1μg était refait à un mois post opératoire objectivant encore l’insuffisance surrénalienne avec une cortisolémie à 103.2ng/ml à 30 minutes et à 98.95ng/ml à 60 minutes. La conduite était de maintenir l’hydrocortisone et on prévoit de répéter ce test à 6 mois après chirurgie.

## Discussion

L’insulinome représente la variété la plus fréquente des tumeurs neuroendocrines du pancréas. Son incidence varie de 1 à 4 cas par million d’habitants et par an [1, 2]. Cette tumeur peut survenir à n’importe quel âge avec une prédilection pour la 5^ème^ décennie. Elle touche de façon presque égale les deux sexes [3-6] avec parfois une prédominance féminine [2, 3]. L’insulinome représente 70 à 80% des tumeurs neuroendocrines pancréatiques fonctionnelles. La tumeur est unique et bénigne dans 90% des cas [5]. Dans 10% des cas, elle est multiple et s’intègre souvent dans le cadre des Néoplasies endocriniennes multiples de type I (NEM I) associant des tumeurs neuroendocrines du pancréas, de la parathyroïde, de l’antéhypophyse, de la corticosurrénale, du thymus ou des bronches. Les circonstances de découverte de l’insulinome sont variées chez le même sujet d’une crise à une autre et différentes d’un malade à un autre [7]. Elles sont à type de signes adrénergiques, troubles de comportement, convulsions, perte de conscience voire même une découverte fortuite. La survenue de malaises souvent graves, le matin à jeun, après un effort, à distance des repas est évocateur d’insulinome [8]. Le diagnostic de l’insulinome est souvent retardé, même chez des patients ayant des malaises fréquents ou chez certains patients présentant pendant plusieurs années des signes neurologiques ou psychiatriques divers [9]. En effet, dans une étude portant sur plusieurs cas d’insulinome, Nathalie C et Marie L. trouvent un délai diagnostique variant de 4 mois à 10 ans [10]. Pour notre patient le délai diagnostic était de 5 ans. Le diagnostic positif se base sur la mise en évidence d’hypoglycémie par une méthode fiable (glycémie sur plasma veineux et non pas glycémie capillaire). En l’absence d’hypoglycémie spontanée, on a recours à l’épreuve de jeun qui reste le gold standard pour confirmer les hypoglycémies. Le diagnostic de l’hyperinsulinisme se base sur le dosage une insulinémie supérieure ou égale à 3μUI/ml concomitante à une hypoglycémie.

L'indice de Turner est considéré comme normal s'il reste inférieur à 50 alors qu’il est en faveur d’un insulinome s’il est supérieur à 200. La confirmation de l’origine endogène de l’hyperinsulinisme se base sur le dosage du peptide C qui a un intérêt dans l’évaluation de la capacité sécrétoire des cellules béta. Donc chez un patient ayant présenté une triade de Whipple, une glycémie <0,5 g/L (spontanément ou lors d’une épreuve de jeûne) concomitante à une insulinémie > 3mUI/L et un peptide C > 0,6ng/mL confirme le diagnostic d’insulinome (si on élimine la prise de sulfamide ou la présence d’Ac anti-insuline). Déterminer la topographie de l’insulinome constitue l’étape à la fois la plus importante puisqu’elle conditionne le geste chirurgical, et la plus difficile du fait de la petite taille de la tumeur et de son faible contraste par rapport au parenchyme pancréatique normal. Le rendement diagnostique des examens paracliniques est variable.

L’échographie abdominale a une sensibilité médiocre allant de 0 à 39%. Ceci est expliqué par le siège profond de la glande pancréatique et l’interposition des gaz digestifs. L’échographie montre généralement un nodule hypoéchogène, homogène, bien limité [11]. La tomodensitométrie (TDM) conventionnelle a une sensibilité comparable à celle de l’échographie, variant de 22 à 43% [5, 6]. Le scanner hélicoïdal est un peu plus performant avec une sensibilité variant entre 15 et 64% [3, 4, 6]. Cette sensibilité passe de 40% pour les lésions de 3cm à 21% seulement pour les tumeurs de moins de 1cm [3]. Une nette amélioration a été récemment apportée par le scanner hélicoïdal biphasique qui, grâce à sa haute résolution et aux coupes fines qu’il offre, permet de détecter la tumeur au cours de la phase artérielle, pendant laquelle le contraste avec le parenchyme sain est optimal. Sa sensibilité atteint ainsi les 94% [5]. La sensibilité de l’IRM est supérieure aux 2 examens précédents pour la détection des tumeurs de petite taille. Elle varie de 85 à 95%. L’insulinome se présente généralement comme une formation en hyposignal en T1 avec rehaussement intense après injection de gadolinium, et en hypersignal en T2 [11]. L’échoendoscopie est considérée par plusieurs auteurs comme l’examen de référence pour la localisation préopératoire de l’insulinome, avec une sensibilité de l’ordre de 80 à 93% [12]. Elle permet de détecter même des tumeurs de petite taille de l'ordre de 5mm [2].

Le traitement de choix est la chirurgie. Un traitement médical peut être prescrit chez un groupe de patients inopérables, lorsque la tumeur est non résécable ou si la maladie est métastatique [13]. Le traitement médical consiste à traiter et à prévenir les hypoglycémies. Le traitement le plus efficace pour le contrôle des hypoglycémies est le diazoxide (50-600 mg/j) qui permet par une action directe sur les cellules bêta de supprimer la sécrétion d’insuline et de déclencher la glycogénolyse [13]. Pour le traitement chirurgical, la technique de choix est l’énucléation mais ce geste n’est pas toujours réalisable du fait de la taille de la tumeur ou de ses éventuels rapports intimes avec le canal de Wirsung, le cholédoque, les vaisseaux spléniques, les vaisseaux mésentériques supérieurs ou le tronc porte. Dans ces cas, une résection pancréatique s’impose. Il s’agit le plus souvent d’une pancréatectomie caudale ou corporéo-caudale avec ou sans conservation splénique, exceptionnellement une duodénopancréatectomie céphalique. La mortalité est faible ne dépassant pas les 2% [1]. Le pronostic dépend essentiellement du degré de malignité de la tumeur qui peut s’avérer souvent difficile à affirmer en l’absence de métastases. Après une résection chirurgicale à visée curative d’un insulinome, une surveillance clinique, biologique et radiologique prolongée s’impose du fait du risque de métastases hépatiques et de récidive locale sur le pancréas restant, éventualité qui reste élevée en cas de tumeur initialement jugée maligne [13].

L'association insulinome et insuffisance antéhypophysaire était décrite dans la littérature mais le mécanisme physiopathologique reste toujours mal élucidé. En effet, une sécrétion de l’hormone de croissance (GH) et cortisol basses et inappropriées à un état d’hypoglycémie sont rapportées dans plusieurs études. Ceci peut conduire à une errance diagnostique quant à l’étiologie de l’hypoglycémie, laissant suspecter à tort une insuffisance corticotrope ou surrénalienne, en particulier lorsque l’image pancréatique d’insulinome n’est pas visible comme le cas de notre patient. La physiopathologie de ce défaut de contre-régulation reste toujours mal élucidée, mais plusieurs études suggèrent le rôle principal de l’hypoglycémie récurrente et de l’hyperinsulinisme chroniques dans le défaut de réponse du cortisol et de la GH à l’hypoglycémie [14].

Concernant l’insuffisance corticotrope l’hypothèse d’une atteinte centrale a été suggérée puisque les concentrations plasmatiques d’ACTH étaient à la limite inférieure de la normale. Des études de biologie moléculaire rapportent que l’hypoglycémie récurrente et l’hyperinsulinisme agiraient au niveau du système nerveux central en élevant le niveau de transcription du gène du récepteur aux glucocorticoïdes [14]. La réversibilité des anomalies hormonales est l’évolution habituelle après chirurgie de l’insulinome et disparition de l’état d’hyperinsulinémie [14]. Dans l’étude de Mitrakou *et al.* [15], la normalisation de la réponse du cortisol et de la GH à l’hypoglycémie est obtenue pour tous les patients dans un délai de trois à six mois après la cure chirurgicale. Malabu *et al.* rapporte le cas d’insulinome associé à un hypogonadisme hypergonadotrope et suggère une hypothèse que des lésions testiculaires irréversibles se constituent suite aux hypoglycémies récurrentes et prolongées, avec des études sur le modèle animal confirmant l'infarctus testiculaire causé par l’hypoglycémie aiguë [16]. De plus, il a été longtemps établi que le dysfonctionnement testiculaire, l'impuissance et l’hypofertilité sont plus fréquents chez les diabétiques de type 1, présentant un risque plus élevé d‘hypoglycémie que ceux ayant un diabète de type 2.

## Conclusion

L’insulinome pancréatique est une tumeur neuroendocrine rare, souvent bénigne, mais qui peut mettre en jeu le pronostic vital du fait des accidents hypoglycémiques qu’elle engendre. Sa prise en charge s’affronte encore à plusieurs difficultés. En effet, son diagnostic se fait encore à un stade tardif surtout chez le sujet âgé. Le diagnostic topographique est jusque-là difficile. Il fait recours à plusieurs techniques dont la sensibilité est variable selon les études. L’écho endoscopie représente actuellement l’examen de référence. En cas d’hyperinsulinisme endogène avec scanner pancréatique normal il faut systématiquement compléter par une écho-endoscopie même si on trouve une insuffisance surrénalienne qui peut expliquer ces hypoglycémies. La physiopathologie de l'insuffisance corticotrope en cas d'insulinome reste toujours mal élucidée, mais plusieurs études suggèrent le rôle de l’hyperinsulinisme chroniques dans le défaut de réponse du cortisol à l’hypoglycémie, une anomalie qui peut être réversible après traitement de l'insulinome. De même les hypoglycémies récurrentes et prolongées peuvent causer un hypogonadisme hypergonadotrope par des lésions testiculaires irréversibles.

## Conflits d’intérêts

Les auteurs ne déclarent aucun conflit d'intérêts.
